# Measuring organizational readiness for implementing change (ORIC) in a new midwifery model of care in rural South Australia

**DOI:** 10.1186/s12913-021-06373-9

**Published:** 2021-04-20

**Authors:** Pamela Adelson, Rachael Yates, Julie-Anne Fleet, Lois McKellar

**Affiliations:** 1 Rosemary Bryant AO Research Centre, UniSA Clinical & Health Sciences, University of South Australia, City East Campus, Playford Building P4-27, North Terrace, Adelaide, SA 5000 Australia; 2 Rural Support Service, South Australia Health, Government of South Australia, Mount Gambier Health Service, Mount Gambier, SA 5290 Australia; 3 UniSA Clinical & Health Sciences, University of South Australia, City East Campus, Playford Building P4-27, North Terrace, Adelaide, SA 5000 Australia

**Keywords:** Organizational readiness, ORIC, Continuity of care, Midwifery, Workforce, Rural and remote, Australia

## Abstract

**Background:**

The sustainability of Australian rural maternity services is under threat due to current workforce shortages. In July 2019, a new midwifery caseload model of care was implemented in rural South Australia to provide midwifery continuity of care and promote a sustainable workforce in the area. The model is unique as it brings together five birthing sites connecting midwives, doctors, nurses and community teams. A critical precursor to successful implementation requires those working in the model be ready to adopt to the change. We surveyed clinicians at the five sites transitioning to the new model of care in order to assess their organizational readiness to implement change.

**Methods:**

A descriptive study assessing readiness for change was measured using the Organizational Readiness for Implementing Change scale (ORIC). The 12 item Likert scale measures a participant’s commitment to change and change efficacy. All clinicians working within the model of care (midwives, nurses and doctors) were invited to complete an e-survey.

**Results:**

Overall, 55% (56/102) of clinicians participating in the model responded. The mean ORIC score was 41.5 (range 12–60) suggesting collectively, midwives, nurses and doctors began the new model of care with a sense of readiness for change. Participants were most likely to agree on the change efficacy statements, “People who work here feel confident that the organization can get people invested in implementing this change and the change commitment statements “People who work here are determined to implement this change”, “People who work here want to implement this change”, and “People who work here are committed to implementing this change.

**Conclusion:**

Results of the ORIC survey indicate that clinicians transitioning to the new model of care were willing to embrace change and commit to the new model. The process of organizational change in health care settings is challenging and a continuous process. If readiness for change is high, organizational members invest more in the change effort and exhibit greater persistence to overcome barriers and setbacks. This is the first reported use of the instrument amongst midwives and nurses in Australia and should be considered for use in other national and international clinical implementation studies.

## Background

The Australian government’s Strategic Directions for Australian Maternity Services [1] highlights the need to maintain and expand existing maternity services in rural and remote Australia. More than half of rural maternity units have closed since 1992 [2, 3] and the sustainability of existing rural services is under threat due to maternity workforce shortages. These shortages have led to the belief that this makes birthing ‘unsafe’ and unviable in rural and remote communities [2]. Closing maternity services has had significant consequences for women and communities, with resulting poorer health outcomes and financial and social hardships [2]. With about 30% of Australian birthing women living in rural and remote areas, there is an outstanding demand for pregnancy, birth and postnatal health services in these areas [4]. Challenges to providing these services include the geographic spread, low population density, recruitment and retention difficulties for midwifery and medical staff and high costs of service delivery [5].

An option for increasing the sustainability of birthing services in regional and rural Australia is implementing midwifery services models such as a midwifery caseload [5]. In most Australian rural and regional settings midwifery care is mostly provided in a traditionally rostered hospital arrangement, whereby midwives are required to work across the role of nurse and midwife [6]. Midwifery caseload is a maternity continuity of carer model whereby care is provided by a known midwife or a secondary backup through pregnancy, birth and the postnatal period, and with assistance from doctors where needed in the event of identified risk factors [7]. High level evidence from trials and multiple studies have demonstrated the benefits and significance of midwifery-led care in terms of maternal satisfaction, efficacy and decreased cost to health services [8–11].

Against this background, a new midwifery continuity of care service model, *Midwifery Caseload Model of Care (MoC) Pilot in Yorke and Northern (Y&N) Region* [12] was designed in collaboration with midwives, nurses, and doctors, including general practitioners (GP) and obstetricians with the aim to ensure a sustainable midwifery workforce in one region of rural South Australia (SA). This MoC provides each woman with a known midwife or a team of midwives to provide care throughout her pregnancy, birth and up to six weeks after birth. The Y&N region was chosen because, while some birthing units in the region were experiencing critical midwifery workforce shortages, others provided successful team and group practice midwifery, providing an opportunity for further development [12]. Collaborations with midwifery and GP/obstetrician workforce focussed on the service delivery model, prioritising choice and interdisciplinary care an important consideration when developing new models of care in maternity services [5, 13, 14].

Considerable community engagement, workshops, and consultation amongst clinicians occurred prior to introducing the MoC as several challenges were anticipated. The MoC brings together five different geographical birthing sites and involves midwives providing continuity of care in partnership with GPs, GP obstetricians, specialist obstetricians, midwives and nurses working at the local hospitals. The existing model required rostering midwives on all shifts at the five rural hospitals and was highly dependent on local doctors to provide shared care, sometimes in areas where the general practitioners were overloaded. While some of these sites had established working relationships amongst clinicians, it was recognised that coming together under one umbrella would require commitment to address potential challenges.

The new MoC would also affect how midwives and nurses operated within the community hospitals. Midwives working in the caseload MoC provide care to women in the community, clinics and in hospitals to support labour and birth and initial postnatal care. As women are provided a primary midwife who is on call, most of the five hospitals would no longer have on-site 24-h midwifery staff. In this model, caring for new mothers and babies was seen as a challenge for some of the nurses accustomed to having onsite midwives in this role.

The Y&N model of care is a two-year pilot program with an independent evaluation utilizing the Proctor framework [15] to assess the implementation of the program. As part of the overall evaluation, it was important to first assess whether clinicians were ready to commit to implementing this significant change. In order for new programs or practices to be successfully implemented in healthcare settings, it is necessary that there is collective support within an organisation to embrace the required change. Several change efforts fail in the health sciences because organisations are not ready or prepared to change [16]. This paper aims to report on readiness for change amongst the midwives, nurses and doctors transitioning to the new model of care.

## Methods

Readiness for change was measured using the Organizational Readiness for Implementing Change scale (ORIC) [16]. The instrument is based on Weiner’s organizational theory [17] and was chosen due to multiple strengths, including; its theory based psychometrically validated measures, measuring readiness for change at the collective level (rather than the individual level) and its brevity for use by busy practitioners [18]. The original English ORIC scale [16] has been translated into several European languages and validated as reliable and valid in health care settings, including those in which nurses, midwives and doctors were participants [18–21].

The 12 item Likert scale ORIC instrument is a robust multilevel construct with a focus on change commitment and change efficacy. Change commitment (5 statements), reflects organizational members’ shared resolve to implement a change and change efficacy (7 items), reflects organizational members’ shared belief in their collective capacity to implement a change [16]. Each of these 12 items is scored using a 5-point Likert scale ranging from “Disagree” to “Agree”. Two additional questions sought the clinician’s primary role and work location.

### Sample

The ORIC survey was timed to coincide with the launch of the new MoC so that clinicians were knowledgeable and aware of the impending change to service delivery, but before the implementation had occurred. The survey was distributed anonymously in August 2019 to 102 clinicians working directly in the MoC or those impacted by the changes; midwives (*n* = 12) and doctors (*n* = 10) transitioning to the model of care, and to midwives and nurses providing direct maternity care at the 5 local hospitals (*n* = 80). Participation was encouraged, but voluntary, and distributed electronically via the survey software SurveyMonkey©. An information sheet was attached to the ORIC instrument and included a statement of implied consent for those completing the survey. A printed version of the questionnaire was also available to clinicians who were unable to access the online survey at work.

### Data analysis

Descriptive analyses were used to describe respondent characteristics and overall ORIC scores. Cronbach’s alpha was used to assess internal consistency and reliability of the scales. Differences between clinician groups scores were examined by one-way analysis of variance (ANOVA) with level of significance specified at 0.05. Analyses were performed with STATA v14.0 (College Station, TX).

## Results

The overall response rate to the survey was 54.9% (56/102) and varied by clinical role. The 12 midwives transitioning to the MoC completed the survey (12/12, 100%), (excludes *n* = 2 midwives who were away and *n* = 1 not yet employed). The response rate of midwives/nurses working at the five local hospitals was 50% (40/80), and for doctors 30% (3/10). One respondent did not answer the two questions regarding location of work and clinical role and disagreed (score of 12) to all statements. Response rates were relatively proportional to the five areas served. Notably, hospital/area 5 represented over a third of all responses, but is also the largest of the five community hospitals (Table 1).

**Table 1:** Clinical role, distribution and mean ORIC scores of participants, *n* = 56

Variables	N	(%)	ORIC score^*^ Mean (SD)
*Clinical Role*			
MoC midwife	12	(21.4)	40.5 (9.5)
Midwife/nurse working in hospital	13	(23.2)	44.5 (11.1)
Nurse working in hospital	27	(48.2)	41.8 (14.1)
Doctor (GP, obstetrician)	3	(5.4)	40.0 (14.7)
Not stated	1	(1.8)	–
*Hospital/primary location of work*			
Hospital/area 1	8	(14.3)	
Hospital/area 2	8	(14.3)	
Hospital/area 3	16	(28.6)	
Hospital/area 4	7	(12.5)	
Hospital/area 5	20	(35.7)	
Not stated	1	(1.8)	

^*^ANOVA *F* 0.26, p = 0.86

Results of the Cronbach’s alpha test demonstrated an overall scale reliability coefficient of 0.96, indicating excellent scale internal consistency quality. The subscale Cronbach’s alpha coefficients for change efficacy was 0.94, and for change commitment 0.90, indicating good reliability.

**Fig. 1:**
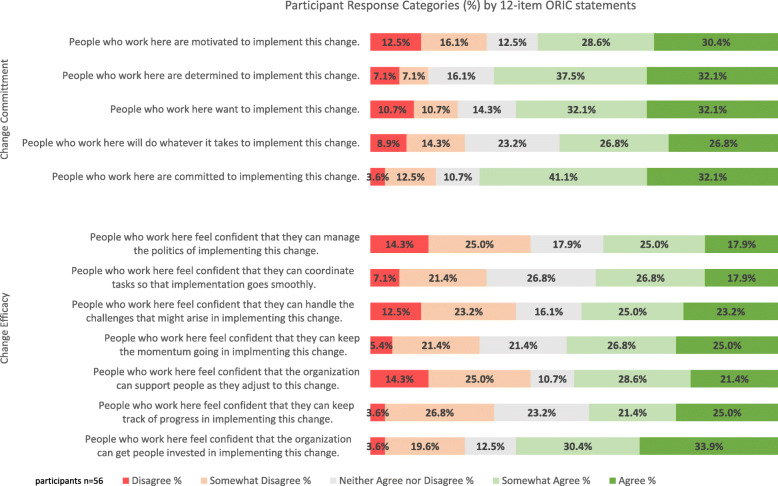
Participant response categories (%) by 12-item ORIC statements

Overall, participants had a mean ORIC score of 41.5 (range 12–60) which suggests collectively, midwives, nurses and doctors have begun the new MoC with a sense of readiness for change. Participants were most likely to agree (33.9%) on the change efficacy statement, “People who work here feel confident that the organization can get people invested in implementing this change”, and the change commitment statements; “People who work here are determined to implement this change (32.1%)”, “People who work here want to implement this change” (32.1%), and “People who work here are committed to implement this change” (32.1%). Participants were most likely to disagree with the change efficacy statements (14.3% for each), “People who work here feel confident that the organization can support people as they adjust to this change” and “People who work here feel confident that they can manage the politics of implementing this change”. Participant responses to all 12 ORIC statements grouped by subscales are shown in Fig. 1. There was no statistically significant difference in the mean ORIC scores as assessed by ANOVA between the professional groups; MoC midwives, doctor, hospital nurse and hospital midwife (*F* 0.26, *p* = 0.86). Bartlett’s test for equal variance was χ^2^ (3df) =2.49, *p* = 0.47 (Table 1).

## Discussion

The evidence for midwifery models of care is well documented and it has been noted that to bridge the gap in translating the evidence to clinical practice in Australia will require widespread reorganisation of the way maternity services are provided [7]. However, it has been observed that systems for designing rural services in Australia do not often use a caseload model strategically to manage the lower numbers that exist in dispersed populations [13].

A critical element of widespread reform with midwifery continuity of carer models is effective collaboration with obstetricians, general practitioners and other medical professions involved in the care of pregnant women [7, 22]. This occurred at an early stage in the development of the MoC with a project team and a development committee that agreed on the terms of reference and were clear in the communication to reduce anxiety and fear of a future change and to explore what was possible with all stakeholders. Within the model of care, graduate midwives are also included and supported through a transition to professional practice program, an important core experience for emerging midwives and a significant factor in succession planning [22]. This collaborative approach embraces a key priority area of the regional strategy; helping clinicians to work together and supporting the attraction and retention of staff for sustainable service well into the future.

Results of the ORIC survey indicate that clinicians transitioning to the new MoC were willing to embrace change and committed to implementing the new model. The process of organizational change in health care settings is challenging and a continuous process. In the case of the new MoC, the proposed changes involved extensive consultation with maternity care providers and the community over a period of approximately 14 months, with clear documentation of how the model would work. The change was viewed as core business in order for a maternity service to survive in the region. The benefits to women, clinicians, and the community were clearly articulated, and this was successful in getting critical buy-in to the MoC, although not without some resistance. It has been proposed that activities such as communicating the important need and urgency for change, and that the benefits are likely to produce changes that matter to stakeholders, are important strategies in preparing for organizational change [23]. Whilst readiness to implement change is only one factor in successfully introducing improvements to a maternity service, readiness to learn and embrace change at various levels within a maternity service has an impact on whether successful implementation of best practice is achieved [24].

Readiness for change is a complex multi-dimensional construct including psychological and structural factors that occur at both the individual and organizational level and requires both a willingness and capability to change [25]. One of the strengths of the ORIC instrument is the measurement of the readiness to change at the unit level rather than the individual level. For successful implementation to the new MoC, those working in the model must be willing and ready to adopt to the change; this has been described as a critical precursor to successful implementation [26]. Assessing readiness for change adds an important component to the MoC evaluation methodology.

Service adaption and innovation is a core strategy of the Australian Government’s Stronger Rural Health Strategy and South Australia’s Rural Health Workforce Plan to promote rural health service sustainability [2]. Change can be very challenging at any time but especially in teams or communities where they don’t see a need to change. It was important to address this as an issue and a risk. It was imperative to bring teams on the discovery, valuing and considering feedback at every milestone and hence we then knew we needed to ‘test’ the readiness for change.

The evidence for whether readiness for change predicts change adoption is an area of great interest and the ORIC instrument has been noted as a scale with promising psychometric properties, but has yet to be tested for predictive validity [23]. If readiness for change is high, organizational members invest more in the change effort and exhibit greater persistence to overcome barriers and setbacks [20]. The ORIC survey is but one element of the planned evaluation of the regional midwifery MoC in South Australia. A complete evaluation of the MoC including provider and user (women’s) assessments is underway and will be reported at the conclusion of the two-year study. This will contribute to the evidence regarding the predictive abilities of readiness for change instruments.

Strengths of this study includes the appropriate timing and targeting of the survey to the appropriate clinician groups. We distributed the survey to all members who would be directly affected by the change. It is important that multiple organizational members who will implement and use the program be surveyed to avoid single-source bias, elite bias and champion bias [23]. Limitations to this study include a less than 60% response rate. However mostly this was due to fewer responses from nurses working in the hospital wards, who would be less affected than the primary providers of care.

## Conclusions

We have found few studies utilising the ORIC instrument in an Australian healthcare environment [27] and none that have been used amongst nurses and midwives in Australia. We believe these results increase the generalizability to other Australian change settings where nurses and midwives will be affected. As the ORIC instrument is theory based, has good to excellent reliability, structural validity, is brief, and has known health-care provider validity [23], it is a good and highly relevant tool to consider when clinical organizational change is being planned. In this survey, clinicians collectively demonstrated a sense of readiness for change to a new, rural maternity service model of care that offers collaborative, safe and effective care to women, babies and their families.

## Data Availability

The dataset generated and analysed for this study are available from the corresponding author on reasonable request.

## Abbreviations

ORIC: Organizational Readiness for Implementing Change
MoC: Model of Care
GP: General Practitioner (medical doctor)
